# Improved quality of life in cystic fibrosis patients observed up to 36 months after starting Elexacaftor/Tezacaftor/Ivacaftor treatment

**DOI:** 10.1186/s41687-025-00879-0

**Published:** 2025-05-06

**Authors:** Francesca Buniotto, Gloria Tridello, Antonella De Scolari, Ilaria Meneghelli, Emily Pintani, Sandra Perobelli, Marco Cipolli

**Affiliations:** Centro Fibrosi Cistica, AOUI Verona, Piazzale Aristide Stefani, 1, Verona, 37126 Italy

**Keywords:** Quality of life, CFQ-R, Cystic fibrosis, (Highly effective) modulator, ETI (Elezacaftor-Ivacaftor-Tezacaftor), Long-term treatment benefits

## Abstract

**Background:**

Elexacaftor/Tezacaftor/Ivacaftor (ETI) is a therapy approved for cystic fibrosis (CF) that has given improved clinical outcomes in patients carrying the F508del mutation. There are few published data regarding ETI’s effects on patients’ quality of life (QoL). This study aims to (fill the data gap in current literature by assessing) evaluate the long-term effects of ETI on QoL.

**Methodology:**

A prospective observational study was conducted with thirty-seven severe patients that received ETI for compassionate use (group A), 184 received it for on-label use (group B). All carried one F508del mutation. Patients were assessed using the CFQ-R (Cystic Fibrosis Questionnaire-Revised). The evaluation time-points were pre-treatment (T0), and after 12 (T1) and 24 months (T2); group A was also assessed after 36 months (T3). Twenty-five patients completed 3 years of treatment and 65 patients completed 2 years of treatment, in groups A and B respectively.

**Results:**

At T1, median values for almost all areas of CFQ-R statistically significant increased in group A, particularly Physical Functioning (+ 25.0), Respiratory (+ 22.2) and Health Perception (+ 22.2).The Social Functioning area statistically significant increased at T2 (+ 5.6). At T3, these improvements remained stable. At T1, all areas of CFQ-R statistically significant increased in group B, particularly the Health Perception (+ 22,2) heading. At T2, these improvements remained stable. For both groups, the changes identified at the last follow-up showed no major differences by gender, age or genetic status.

**Conclusions:**

Treatment with ETI significantly improved patients’ QoL in both groups at 12–24 months, these improvements remaining stable in patients tested at 36 months.

**Supplementary Information:**

The online version contains supplementary material available at 10.1186/s41687-025-00879-0.

## Background

Cystic Fibrosis before the advent of the Cystic Fibrosis Transmembrane Conductance Regulator (CFTR) modulators was once considered a chronic, progressively worsening disease, the result of this view being that patients’ life planning was very limited. CFTR modulators acting on the underlying causative mechanism changed the approach to the disease and the life expectancy of patients, as shown in preliminary studies [1].

The last approved modulator Elexacaftor/Tezacaftor/Ivacaftor (ETI) is a triple combination therapy, available in Italy since July 2021 for patients aged 12 years and over with at least one F508del mutation. Improvements in the following parameters were reported as early as 4 weeks of ETI treatment, in studies comparing it with placebo [2] and with Tezacaftor-Ivacaftor alone [3]: Forced Expiratory Volume in 1 s (FEV1); sweat chloride concentrations; pulmonary exacerbations, which were significantly reduced; body mass index (BMI); and the Respiratory Symptoms score on the Cystic Fibrosis Questionnaire-Revised (CFQ-R), a questionnaire used to assess CF patients’ quality of life (QoL).

CFQ-R, as recommended by the Good Clinical Practice guidelines for clinical research and trials [4], is an appropriate QoL questionnaire as it can provide added value in the form of patient-reported data not captured by other endpoints [5, 6].

Few studies to date have considered ETI effects on patients’ QoL as measured by the CFQ-R. Some studies have reported QoL data specifically related to the Respiratory Symptoms Domain (RD) [2, 3]. Other research covering all CFQ-R domains (RD/non-RD) has mainly reported short-term results [7, 8], only one study showing ETI effects at 24 months [9].

This study aimed to fill the data gap in current literature by assessing evaluate the long-term effects of ETI on QoL. This was a prospective observational study conducted at the Cystic Fibrosis Center in Verona. The study focused on CF patients with at least one F508del mutation, treated with a CFTR modulator (ETI). These patients were evaluated at different time-points, in relation to clinical parameters (FEV1, BMI, growth data, liver function, sweat test, 6MWT) and QoL, assessed by the CFQ-R.

Here, we report as primary findings QoL data for all domains after 12, 24 and 36 months of treatment, as a secondary findings we analyze the results according to patients’ gender, age and genetic mutations.

## Methodology

### Study design and eligibility criteria

At the CF Center in Verona, prescription of ETI began in October 2019 for compassionate use in patients with severe CF, aged ≥ 12 years (**group A**, 37 pts). All these patients were homozygous for F508del, or heterozygous for F508del with minimal function mutation.

Inclusion criteria were: FEV1 < 40% predicted for a minimum of 2 months; and/or ongoing evaluation for a lung transplant, or inclusion on the waiting list for one.

Exclusion criteria were any one of the following: ongoing invasive mechanical ventilation; severe hepatic insufficiency; history of advanced liver disease; history of solid organ transplantation or hematopoietic cell transplantation; history of alcohol or drug abuse; ongoing pregnancy.

All patients (or parents of minors) were informed of the study’s aims and procedures, and their written informed consent was obtained. The study was approved by the regional Ethics Committee of Regione Veneto (N. 36003 study group A; N. 2858CESC study group B).

### Study design

This is a single-center observational study. The protocol required evaluations at the following timepoints: baseline (T0); 1, 3, 6, 12 months (T1), 24 months (T2); and, for group A, also 36 months (T3) after the beginning of treatment. Here, we consider only the long-term results at 12, 24 and 36 months. At each timepoint, clinical and QoL data were collected. The CFQ-R was used, investigating 12 areas of life (emotional functioning, social functioning, role functioning, treatment burden, weight, physical functioning, perception of health, digestive symptoms, nutrition, body image, weight, vitality) and scoring the various items (0-100). The CFQ-R, created specifically to assess QoL in patients with CF, currently exists in 4 versions for different categories of respondents: children aged 6 to 11 years; those aged 12–13 years; parents of children/adolescents aged 6 to 13 years; adolescents-adults, aged *≥* 14 years [5, 6]. There are validated translations of the questionnaire in many languages, including Italian [10]. It is a self-administered questionnaire, taking about 15 min to complete. The initial part is concerned with personal data (age, gender, nationality, marital status, level of education, occupation). This is followed by 50 items with 4 possible responses, rated on a 4-point Likert scale, which measure the intensity (very difficult, some difficult, little difficult, no difficult) or frequency of a certain behavior or mood (always, often, sometimes, never; very, quite, very little, very little; very, moderately, little, not at all), as well as a true-false scale for some items (completely true, quite true, quite false, completely false). Standardized scores range from 0 to 100, where higher values are indicative of a better QoL. Scores are grouped in 12 areas: 6 of these headings are generic (Health Perception, Physical Functioning, Vitality, Role Functioning, Emotional Functioning, Social Functioning), while 6 are specific for CF (Body Image, Eating Problems, Weight, Treatment Burden, Respiratory and Digestive Symptoms). The questionnaire focuses on the patient and how s/he perceives his/her overall functioning and well-being; patients are asked to express how they have felt over the past two weeks, both physically and emotionally, in the various areas described above. The Respiratory Symptoms area of the questionnaire is often used as an outcome measure in clinical trials to evaluate the efficacy of a drug [11], but all the scales can be used independently.

### Statistical analysis

Patients’ characteristics were reported by descriptive statistics: median, minimum and maximum values were used for the continuous variables, absolute and percentage frequencies for categorical variables. Baseline data were compared with 12-month, 24-month and 36-month values by using the Wilcoxon signed-rank test. The Mann-Whitney test was used to test differences according to sex and age: a p-value < 0.05 was considered statistically significant. For all analyses, the software used was SAS v 9.4.

## Results

Of the 37 patients, 5 were excluded from this analysis (4 because of incomplete responses to CFQ-R, 1 because of a transplant procedure). Of the remaining 32 patients, 25 completed 3 years of treatment. Following AIFA (Italian Medicines Agency) approval, until 31/08/2023 184 patients aged *≥* 12 years were enrolled (**group B**): all of them carried at least one F508del mutation. Of these, 162 patients over 14 years completed 1 year of treatment. Since 13 patients were excluded from this analysis (9 because of incomplete responses to CFQ-R, while 4 discontinued treatment due to adverse effects), 149 patients remained eligible for analysis at 1 year; 65 of these also completed 2 years of treatment.

Table 1 shows demographic and clinical characteristics, with baseline, 12- and 24-month CFQ-R scores for both group A (compassionate use) and group B (on-label use). For group A, 36-month scores are also shown. (See Table 1A in the Online Resource 1 for further information.)

**Table 1:** Demographic and clinical characteristics and CFQ-R scores, at different timepoints, in groups A and B

Characteristics	Group A					Group B			
	T0 (*N* = 32)	T1 (*N* = 32)	T2 (*N* = 31)	T3 (*N* = 25)	T0-T3 (*N* = 25)	T0 (*N* = 149)	T1 (*N* = 149)	T2 (*N* = 65)	T0-T2 (*N* = 65)
Sex M/F (n)	15/17					72/77			
Age (median, min-max)	39.7 (18.0–52.0)					28.3 (13.9–58.5)			
FEV1% (median, min-max)	37.0 (21.0–55.0)	46.0 (28.0–96.0)	49.0 (27.0–89.0)	48.0 (31.0–83.0)	12.5* (0.0–34.0)	70.0 (27.0-112.0)	87.0 (29.0-127.0)	75.5 (41.0-126.0)	13.0* (3.0–45.0)
BMI (median, min-max)	20.8 (15.6–27.1)	22.5 (17.9–29.3)	22.2 (17.8–31.6)	22.0 (17.4–26.2)	1.3* (-0.7-3.7)	20.5 (15.3–26.8)	21.6 (16.0-27.4)	21.9 (16.6–26.5)	1.2* (-2.4-5.1)
CFQ-R (median, min-max)									
Physical Functioning	50.0 (0.0-95.8)	85.4 (25.0-100.0)	79.2 (29.2–100.0)	75.0 (33.3–100.0)	29.2* (-37.5-79.2)	75.0 (8.3–100.0)	91.7 (12.5–100.0)	87.5 (25.0-100.0)	16.7* (-20.8-91.7)
Vitality	58.3 (8.3–83.3)	75.0 (41.7–100.0)	66.7 (41.7–100.0)	75.0 (41.7–100.0)	16.7* (-8.3-66.7)	58.3 (0.0-100.0)	75.0 (0.0-100.0)	75.0 (16.7–100)	16.7* (-41.7-83.3)
Emotional Functioning	73.3 (40.0-100.0)	80.0 (46.7–100.0)	80.0 (46.7–100.0)	86.7 (46.7–100.0)	6.7* (-13.3-33.3)	73.3 (0.0-100.0)	86.7 (13.3–100.0)	86.7 (26.7–100)	6.7* (-33.3-73.3)
Eating Problems	100.0 (33.3–100.0)	100.0 (55.6–100.0)	100.0 (77.8–100.0)	100.0 (77.8–100.0)	0.0* (0.0-66.7)	100.0 (0.0-100.0)	100.0 (33.3–100.0)	100 (44.4–100.0)	0.0* (-11.1-77.8)
Treatment Burden	44.4 (0.0-77.8)	55.6 (0.0-88.9)	55.6 (22.2–88.9)	55.6 (33.3–88.9)	11.1* (0.0-44.5)	44.4 (0.0-100.0)	66.7 (11.1–100.0)	66.7 (22.2–100.0)	11.1* (-22.2-88.9)
Health Perception	44.4 (0.0-88.9)	72.2 (33.3–100.0)	77.8 (44.4–100.0)	66.7 (44.4–100.0)	22.2* (-22.2-77.8)	66.7 (0.0-100.0)	77.8 (0.0-100.0)	77.8 (33.3–100.0)	22.2* (-22.2-100.0)
Social Functioning	66.7 (22.2–94.4)	66.7 (33.3–94.4)	77.8 (50.0-94.4)	83.3 (44.4–100.0)	11.1* (-16.7-33.3)	66.7 (0.0-100.0)	77.8 (16.7–100.0)	77.8 (33.3–100.0)	16.7* (-27.7-88.9)
Body Image	77.8 (22.2–100.0)	94.4 (33.3–100.0)	88.9 (33.3–100.0)	88.9 (0.0-100.0)	0.0 (-33.3-55.6)	77.8 (0.0-100.0)	77.8 (0.0-100.0)	88.9 (33.3–100.0)	11.1* (-22.2-100.0)
Role Functioning	75.0 (0.0-100.0)	91.7 (33.3–100.0)	91.7 (50.0-100.0)	91.7 (50.0-100.0)	16.7* (-33.3-66.7)	83.3 (16.7–100.0)	91.7 (25.0-100.0)	91.7 (50.0-100.0)	8.3* (-16.7-75.0)
Weight	66.7 (0.0-100.0)	100.0 (0.0-100.0)	100.0 (33.3–100.0)	100.0 (0.0-100.0)	33.3* (-66.7-100.0)	66.7 (0.0-100.0)	100.0 (0.0-100.0)	100.0 (0.0-100.0)	33.3* (-33.4-100.0)
Respiratory Symptoms	72.2 (16.7–88.9)	94.4 (27.8–100.0)	88.9 (66.7–100.0)	94.4 (72.2–100.0)	22.2* (-5.6-44.4)	72.2 (11.1–100.0)	94.4 (27.8.-100.0)	94.4 (38.9–100.0)	22.2* (-33.3-88.9)
Digestive Symptoms	88.9 (33.3–100.0)	88.9 (44.4–100.0)	88.9 (55.6–100.0)	88.9 (55.6–100.0)	0.0 (-22.2-44.5)	77.8 (0.0-100.0)	77.8 (22.2–100.0)	77.8 (33.3–100.0)	0.0 (-33.4-55.6)

CFQ-R: Cystic Fibrosis Questionnaire-Revised; Fev1%: Forced Expiratory Volume in 1 s %; BMI: Body Mass Index

Group A: patients treated with ETI before Italian Medicines Agency approval

Group B: patients treated with ETI after Italian Medicines Agency approval

* *p* < 0.05

**Fig. 1:**
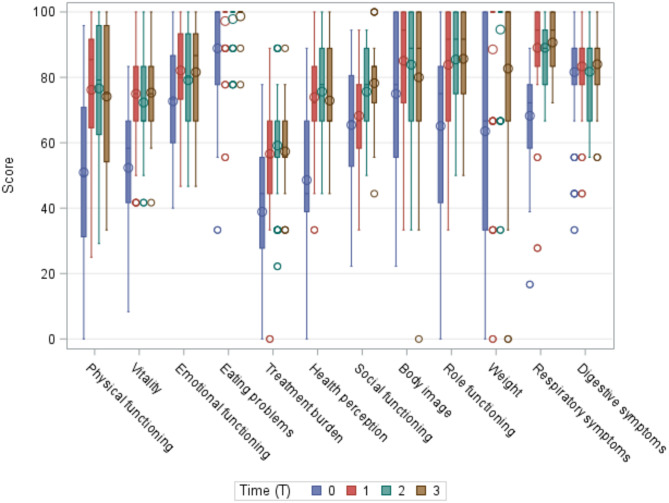
Group A: CFQ-R scores at different timepoints (T0 = baseline, T1 = 1 year, T2 = 2 years, T3 = 3 years)

**Fig. 2:**
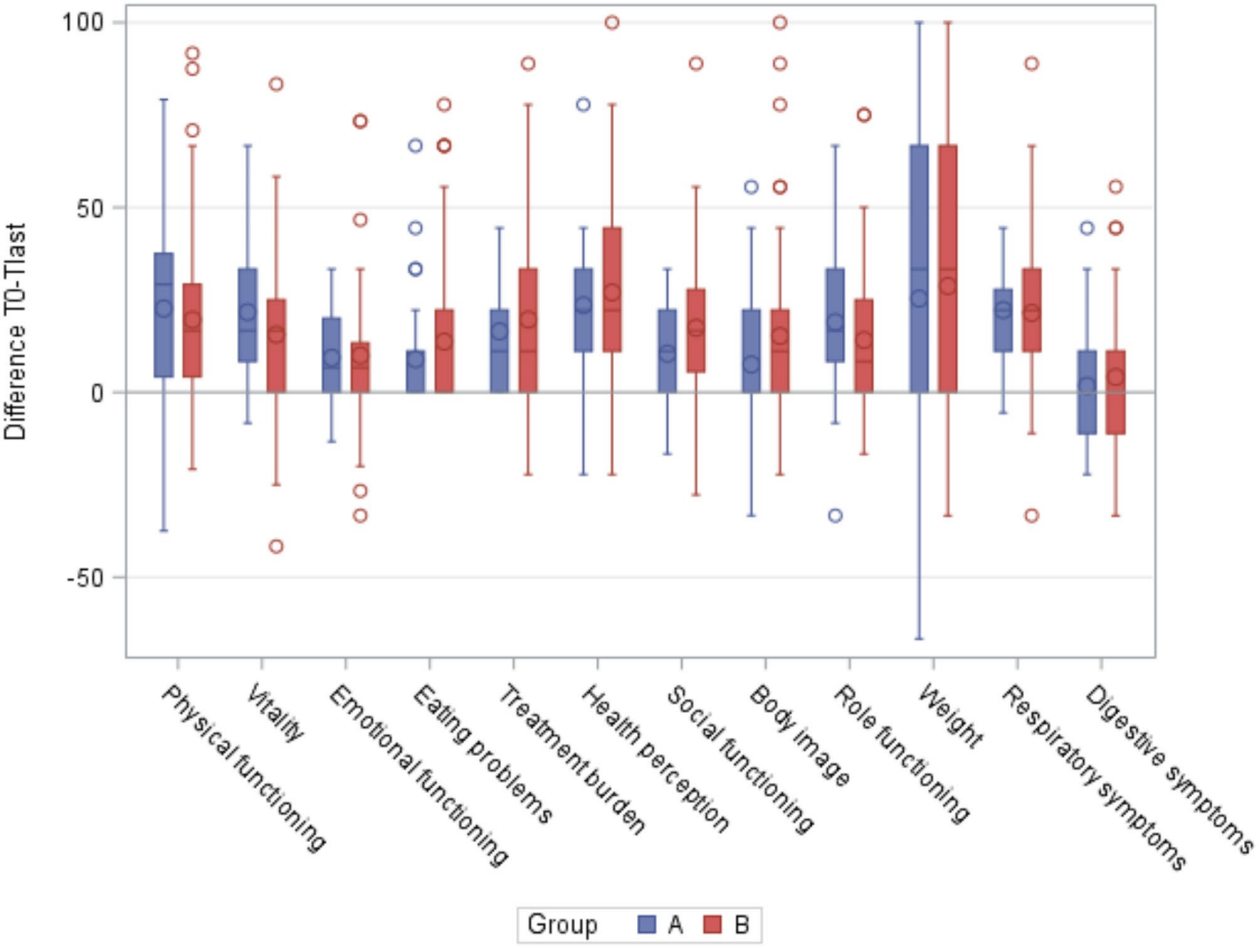
Differences in CFQ-R scores (T0-Tlast)

At baseline, group A had a median age of 39.7 years (min-max = 18.0–52.0), severe respiratory conditions and a low median FEV1 (37.0 median; min-max = 21.0–55.0). These clinical features impact particularly on the Physical Functioning, Health Perception and Role Functioning areas of the CFQ-R. In group A, the greatest statistically significant improvements in CFQ-R scores occurred in the first year of treatment. The most noteworthy of these results were found in Physical Functioning (median + 25.0), Respiratory Symptoms (median + 22.2), Health Perception (median + 22.2), Vitality (median + 16.7), Role Functioning (median + 16.7) and Treatment Burden (median + 11.1). These values remained quite stable at 24 and 36 months, while the Digestive Symptoms score remained quite problematic (median differences = 0.0). See Figs. 1 and 2.

**Fig. 3:**
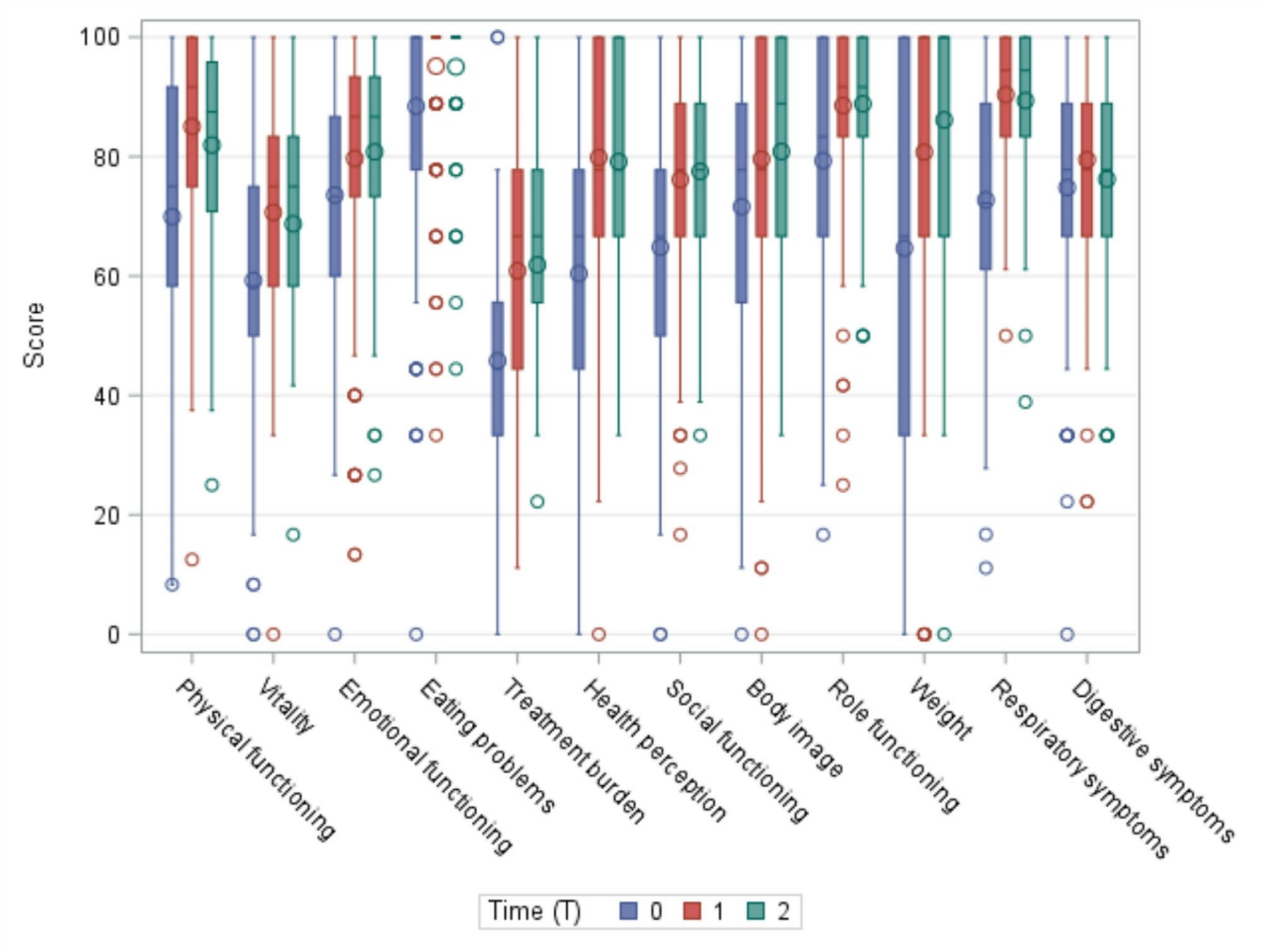
Group B: CFQ-R scores at different timepoints (T0 = baseline, T1 = 1 year, T2 = 2 years). Influencing factors: age, gender and genetics

In group B (median age = 28.3 years, min-max = 13.9–58.5), almost all areas of the questionnaire showed a statistically significant increase from baseline after the first year of treatment. This improvement continued in the following months. As in group A, the Digestive Symptoms area (median differences = 0.0) remained quite problematic. After two years of treatment, statistically significant improvements were reported in Health Perception (median + 22.2), Respiratory Symptoms (median + 22.2), Physical Functioning (median + 16.7), Vitality (median + 16.7), Social Functioning (median + 16.7) and Treatment Burden (median + 11.1). See Figs. 2 and 3.

**Table 2A:** Comparison of differences in CFQ-R scores (T0-Tlast^1^), by gender

	Group A			Group B		
	T0-T3			T0-T2		
	M (*N* = 13)	F (*N* = 12)	*p*	M (*N* = 26)	F (*N* = 39)	*p*
FEV1% (median, min-max)	14.0 (9.0–34.0)	10.0 (0.0–26.0)	0.0514	12.0 (4.0–45.0)	17.0 (3.0–36.0)	0.3589
BMI (median, min-max)	1.4 (-0.7-3.7)	1.1 (0.3–2.8)	0.7033	1.2 (-2.4-5.1)	1.2 (-1.9-4.2)	0.7632
CFQ-R (median, min-max)						
Physical Functioning	20.8 (-37.5-58.3)	29.2 (-20.8-79.2)	0.6238	10.4 (-16.7-87.5)	16.7 (-20.8-91.7)	0.7376
Vitality	16.7 (-8.3-66.7)	33.3 (0.0-41.7)	0.1624	25.0 (0.0–50.0)	8.4 (-41.7-83.3)	0.4421
Emotional Functioning	0.0 (-13.3-26.7)	16.7 (-6.7-33.3)	0.0557	6.7 (-26.7-73.3)	6.7 (33.3–73.3)	0.5348
Eating Problems	0.0 (0.0-66.7)	0.0 (0.0-44.4)	0.4920	0.0 (0.0-66.7)	0.0 (0.0-77.8)	0.4449
Treatment Burden	11.1 (0.0-44.5)	22.2 (0.0-44.5)	0.5068	11.1 (-11.1-55.6)	11.2 (-22.2-88.9)	0.8606
Health Perception	22.2 (-22.2-77.8)	27.8 (11.1–44.5)	0.3090	22.2 (0.0-77.8)	22.2 (-22.2-100.0)	0.7824
Social Functioning	0.0 (-16.7-33.3)	13.9 (-5.6-33.3)	0.2996	16.7 (-11.1-38.9)	22.2 (-27.7-88.9)	0.2540
Body Image	0.0 (-33.3-33.3)	22.2* (0.0-55.6)	0.0034	11.1 (-11.1-55.6)	11.1 (-22.2-100.0)	0.9946
Role Functioning	16.7 (-33.3-50.0)	16.7 (0.0-66.7)	0.4430	8.3 (-16.7-75.0)	8.3 (-8.4-75.0)	0.5457
Weight	0.0 (-66.7-66.7)	33.3 (0.0-100.0)	0.0563	33.3 (-33.3-100.0)	33.3 (-33.4-100.0)	0.9778
Respiratory Symptoms	16.7 (-5.6-44.4)	27.8 (11.1–44.4)	0.2388	19.4 (-11.1-55.6)	22.2 (-33.3-88.9)	0.9465
Digestive Symptoms	0.0 (-22.2-44.5)	5.6 (-11.1-33.3)	0.4346	0.0 (-33.4-44.5)	0.0 (-22.2-55.6)	0.8067

^1^Tlast = last timepoint

**Table 2B:** Comparison of differences in CFQ-R scores (T0-Tlast^1^), by age

	Group A			Group B		
	T0-T3			T0-T2		
	< 30 yrs (*N* = 6)	≥ 30 yrs (*N* = 19)	*p*	< 30 yrs (*N* = 34)	≥ 30 yrs (*N* = 31)	*p*
FEV1% (median, min-max)	14.0 (2.0–34.0)	12.0 (0.0–26.0)	0.3662	18.0 (3.0–45.0)	12.0 (3.0–25.0)	0.0462
BMI (median, min-max)	1.9 (-0.2-2.9)	0.9 (-0.7-3.7)	0.2266	1.3 (-0.4-5.1)	1.2 (-2.4-4.2)	0.9529
CFQ-R (median, min-max)						
Physical Functioning	35.4 (0.0-58.3)	20.8 (-37.5-79.2)	0.3077	12.5 (-8.3-91.7)	20.8 (-20.8-66.6)	0.2990
Vitality	33.3* (16.7–66.7)	16.7 (-8.3-41.7)	0.0373	8.3 (-8.4-83.3)	16.7 (-41.7-58.3)	0.6014
Emotional Functioning	6.7 (-13.3-26.7)	6.7 (-6.7-33.3)	0.7492	6.7 (-33.3-73.3)	13.3 (-26.7-73.3)	0.3647
Eating Problems	5.6 (0.0-66.7)	0.0 (0.0-33.3)	0.1267	0.0 (0.0-66.7)	0.0 (0.0-77.8)	0.2385
Treatment Burden	11.1 (0.0-22.3)	11.1 (0.0-44.5)	0.8208	11.1 (-22.2-88.9)	11.2 (-11.1-77.8)	0.5598
Health Perception	27.8 (-22.2-77.8)	22.2 (0.0-44.5)	0.3346	22.2 (-11.1-100.0)	33.3 (-22.2-77.8)	0.7065
Social Functioning	13.9 (-5.6-16.7)	5.6 (-16.7-33.3)	0.7495	16.6 (-27.7-88.9)	22.2 (-11.1-55.6)	0.1903
Body Image	11.1 (-33.3-55.6)	0.0 (-22.2-33.3)	0.3071	11.1 (-22.2-100.0)	11.1 (-22.2-77.8)	0.4816
Role Functioning	29.2 (0.0-41.7)	16.7 (-33.3-66.7)	0.3051	8.3 (-8.4-75.0)	8.3 (-16.7-75.0)	0.6363
Weight	50.0 (0.0-100.0)	0.0 (-66.7-66.7)	0.2175	33.3 (-33.4-100.0)	33.3 (-33.3-100.0)	0.9619
Respiratory Symptoms	41.7 (5.6–44.4)	16.7 (-5.6-38.9)	0.1240	22.2 (-11.1-88.9)	22.2 (-33.3-55.6)	0.9790
Digestive Symptoms	0.0 (-22.2-33.3)	0.0 (-22.2-44.5)	0.6015	0.0 (-22.2-55.6)	0.0 (-33.4-44.5)	0.7744

^1^Tlast = last timepoint

**Table 2C:** Comparison of differences in CFQ-R scores (T0-Tlast^1^), by genetic status

	Group A			Group B		
	T0-T3			T0-T2		
	Heterozygotes (*N* = 15)	Homozygotes (*N* = 10)	*p*	Heterozygotes (*N* = 43)	Homozygotes (*N* = 22)	*p*
FEV1% (median, min-max)	12.0 (2.0–34.0)	14.0 (0.0–26.0)	0.4543	12.5 (3.0–45.0)	14.0 (3.0–29.0)	0.7932
BMI (median, min-max)	1.4 (-0.7-3.7)	0.8 (-0.2-2.9)	0.5790	1.3 (-2.4-5.1)	1.1 (-1.9-3.6)	0.1573
CFQ-R (median, min-max)						
Physical Functioning	29.2 (-37.5-45.8)	25.0 (0.0-79.2)	0.3890	20.8 (-16.7-91.7)	10.4 (-20.8-87.5)	0.2410
Vitality	16.7 (0.0-41.7)	25.0 (-8.3-66.7)	0.5388	16.7 (-8.3-83.3)	8.3 (-41.7-50.0)	0.1465
Emotional Functioning	0.0 (-6.7-26.7)	20.0 (-13.3-33.3)	0.0700	13.3* (-33.3-73.3)	3.3 (-20.0-33.3)	0.0458
Eating Problems	0.0 (0.0-44.4)	0.0 (0.0-66.7)	0.7260	0.0 (0.0-66.7)	0.0 (0.0-77.8)	0.1172
Treatment Burden	11.1 (0.0-44.5)	11.1 (0.0-33.3)	0.9326	22.2 (-22.2-88.9)	11.1 (-11.1-55.6)	0.1017
Health Perception	22.2 (-22.2-44.5)	22.2 (0.0-77.8)	0.3847	33.3* (-11.1-100.0)	16.7 (-22.2-55.6)	0.0101
Social Functioning	5.6 (-16.7-33.3)	16.7 (-5.6-33.3)	0.1815	22.2 (-27.7-88.9)	11.1 (-5.6-38.9)	0.1690
Body Image	0.0 (-33.3-55.6)	0.0 (0.0-22.2)	0.8183	11.1 (-22.2-100.0)	11.1 (-22.2-55.6)	0.3849
Role Functioning	16.7 (-33.3-50.0)	16.7 (0.0-66.7)	0.9777	8.3 (-16.7-75.0)	0.0 (-8.4-50.0)	0.0916
Weight	0.0 (-66.7-100.0)	33.3 (-33.3-66.7)	0.8388	33.3 (-33.4-100.0)	0.0 (-33.3-100.0)	0.2551
Respiratory Symptoms	22.2 (5.6–44.4)	11.1 (-5.6-44.4)	0.1624	22.2 (-11.1-88.9)	16.7 (-33.3-55.6)	0.4161
Digestive Symptoms	0.0 (-22.2-44.5)	0.0 (-22.2-22.2)	0.8421	0.0 (-33.4-55.6)	0.0 (-22.2-33.3)	0.3143

^1^Tlast = last timepoint

As shown in Tables 2A, B and C, for both group A and group B the changes identified at the last follow-up showed no major differences according to gender, age and genetics. Higher scores were observed for Vitality in younger patients in group A (median = + 33.3, min-max = 16.7–66.7, pts < 30 vs. median = + 16.7, min-max= -8.3-41.7 pts > 30, p 0.0373), and for Body Image in group A females (median = + 22.2, min-max = 0.0-55.6 female vs. median = 0.0, min-max= -33.3-33.3 male, p 0.0034).

Homozygous status, reported for 40% of group A patients and 34% in group B, did not influence any areas in group A. In group B, homozygotes showed statistically significant lower scores in Emotional Functioning (median = + 13.3, min-max= -33.3-73.3, heterozygous vs. median = + 3.3, min-max= -20.0-33.3 homozygous, p 0.0458) and Health Perception (median = + 33.3, min-max=-11.1-100.0 heterozygous vs. median = + 16.7, min-max= -22.2-55.6 homozygous, p 0.0101).

Baseline FEV1 (> or < 60%) did not show any effect on CFQ-R scores in group B.

Finally, the correlation between FEV1 or BMI scores and CFQ-R values within each group showed no statistically significant difference.

## Discussion

This paper presents data from a study carried out at the Verona Cystic Fibrosis Center, with two groups of patients: group A were severe patients who took ETI on a compassionate use basis, with a follow-up of 36 months, while group B took on-label ETI, and completed a follow-up of 24 months.

To our knowledge, this is the first study with data regarding the impact of ETI on patients’ QoL after 36 months of treatment, previous studies with QoL data being limited to a period of 3, 6 or 24 months [7–9].

Recently Gruber et al. [12] reported significant improvements in most CFQ-R domains in 21 adult CF patients treated with ETI for 33 ± 25 weeks, compared to a small group of 6 non-ETI patients. While the study described is longitudinal, it should be noted that the first CFQ-R assessment of the participating patients was done in an earlier project that ran from 2014 to 2018, and thus predated the introduction of ETI.

The primary aim of our study demonstrated that ETI therapy showed a positive long-term effect on all aspects of QoL covered by the CFQ-R, even in patients with severe disease. No correlation was found between CFQ-R scores at the baseline and FEV1 or BMI. In addition, after three years of treatment we observed an improvement in terms of Social Functioning (“I don’t have to stay at home more than I want to”, “I feel comfortable discussing my illness with others”, “I get together with my friends a lot”, “I feel comfortable going out at night”). This indicates that the better the patients feel, the more at ease they are with their condition; no longer suffering the fits of coughing that embarrassed them and made them uncomfortable in front of others, they also feel stronger, less tired, and more motivated to go out and socialize.

Probably this delay in the improvement of the Social Functioning area for group A could be related to the major social restrictions caused by the outbreak of the COVID-19 pandemic in 2020.

Though there are no standardized criteria for minimum clinically important differences in non-RD scores [11], the values of these domains exceeded the 4 points applied to RD scores. In this study, the sizeable and lasting upturns in scores indicate what we consider to be a real improvement.

Carrasco Hernandez et al. [9] reported a decline in CFQ-R values after 12 months of treatment; in our study, despite the comparably severe condition of Group A patients (advanced disease, treated with ETI on a compassionate use basis), the improvement of CFQ-R scores was still present at 24 and 36 months.

As reported by previous studies [7, 8, 9], both our groups showed no statistically significant improvement in the Digestive Symptoms area: the same problems persisted also after ETI treatment (diarrhea, abdominal pain and bloating, flatulence). We did not collect data on this aspect, however it has been already established that ETI is less effective in modifying the pre-existing chronic intestinal inflammation and structural tissue damage in adult patients [13]. This may explain the lack of improvement in gastrointestinal (GI) symptoms and their perception of quality of life. Some articles regarding GI symptoms have reported different results in this respect. Mainz et al. [13] observed improved GI symptoms with ETI, using a CF-specific questionnaire (CFAbd-Score). The PROMISE study [14] demonstrated a small but statistically significant improvement in GI symptoms with ETI. Another study [15], based on an electronic survey, reported that GI symptoms affected the CF patients’ QoL and remained prevalent, albeit possibly of a different nature, even in those treated with modulators. Future studies should focus on GI symptoms and QoL also investigating other intestinal aspects such as pancreatic insufficiency.

The secondary aim of this study was to test influencing factors such as genetics, age and gender in QoL.

It is known that PwCF homozygous for F508del generally have a more severe disease and consequently this may influence their QoL [16, 17].

In our study both groups showed an increase in all domains of CFQ-R over 36 months, independently of their genotype. We only noted a smaller increase in Health Perception and Emotional Functioning in homozygous patients of group B, from baseline to the last timepoint. Similar results were shown in the studies by Di Mango et al. [8] and Fajac et al. [7].

On the contrary, Carrasco Hernandez et al. [9] identified a difference between heterozygous and homozygous subjects, with the latter showing a greater decrease in CFQ-R scores after 12 months. The authors did not provide explanation on this result. We think that the persistence of a better clinical status may be the principal factor influencing QoL, regardless of the genetic defect. In general, the perception of QoL depends on many factors, including the acceptance and the adaptation to the disease, family environment and best supportive care [1].

Previous research on ETI and CFQ-R did not investigate gender differences. In our study, a gender difference was found in group A, where the Body Image score of males does not improve despite ETI therapy and a better BMI. Looking at factors that might influence QoL in CF adolescents and adults, Sawicki et al. [18] found that male subjects showed lower Body Image scores than females. A potential explanation for this is given by Abbott et al. [19]: male patients with CF desire a more muscular build and are less satisfied with their body image, whereas female patients wish to be slim (due to cultural stereotypes) and are often satisfied with their lower body weight. In our study, males in group A with low baseline BMI achieved a weight gain but did not perceive this positively, as they gained no muscular bulk. An earlier study [20] suggested worse QoL outcomes in women with CF, outside the Body Image and Weight domains; in our case, we found that women reported higher scores than men in several CFQ-R domains, though these differences were statistically significant only in the Body Image area.

Before ETI, our older patients experienced progressive worsening of health and an increase in extrapulmonary comorbidities, associated with perception of deteriorating QoL. Sawicki and Dill [21, 22] found that increasing age was consistently associated with higher Treatment Burden scores on the CFQ-R. For this reason, we set out to examine the age variable in our study. Hochwälder et al. [23] noted that young adults had higher QoL scores than adults for 4 out of 12 scales, the differences being statistically significant. Our sample was characterized by a wider age range and a greater number of older patients than others reported to date: group A, median 39.7 years (min-max = 18–52 y); group B, median 28.3 years (min-max = 14-58.5 y). ETI was associated with improved Vitality scores in younger patients in group A, despite the severity of their condition. Given the progressive nature of the disease, older patients in more severe condition show smaller improvements in QoL, though there is no statistically significant age-related trend in this respect. By the same token, our study did not identify age as an influencing factor in relation to Treatment Burden: this was probably because ETI improved patients’ health, regardless of age.

With ETI treatment, there have been instances of patients reporting a change in their lives, feeling more hopeful about the future [24]. Consistent with this, our results indicate a general improvement in patients’ QoL, with the areas that show the most marked increase leading to better social and role functioning: patients find it easier to plan for the future (e.g., going to college, getting married, getting promoted at work), and do not feel that CF causes greater limitations in terms of missing school or work, cutting back on socialization, or feeling unable to complete daily activities.

As reported by Gruber et al. [12], our clinical practice too has identified difficulties in adapting to the changes that accompany ETI therapy (e.g., emotional highs and lows, loss of identity, fear of the disease worsening). Such difficulties were observed in a few patients, but did not affect overall CFQ-R values. These problematic areas need to be followed over a period of time by means of appropriate studies.

Di Mango et al. [8] found no correlation between improvement in FEV1 and any of the domains on the CFQ-R, or between BMI and CFQ-R. Our study gave similar results in this respect, identifying no such correlation.

### Limitations of the study

Some limitations of this study must be taken into account. For example, a significant minimum clinically important difference has not been established for non-RD domains in the CFQ-R questionnaire. We hope that this shortcoming, also reported by other authors [7], may be addressed by appropriate research, given the importance of a disease-specific questionnaire like CFQ-R for research in CF.

In our study, there was no control group. However, longitudinal studies of adult CF populations indicate that, given the progressive nature of the disease, QoL does not improve naturally but stabilizes or decreases over time [21, 22].

Our study was conducted in 2020–2021 during the COVID-19 pandemic. The major social restrictions adopted in Italy influenced people’s QoL. This outside factor could have had an impact on some of the QoL answers as observed in the social functioning area of the CFQ-R.

In conclusion, it will be interesting to see the impact of ETI in the pediatric population, for whom studies are still few in number and surveys on QoL changes depend mostly on caregivers.

## Conclusions

ETI treatment significantly improved patients’ QoL in both groups. These improvements were maintained over long-term follow-up (36 months). For both groups, the changes identified at the last follow-up showed no major differences by gender, age or genetic status.

It will be interesting to explore in future research the long-term effects of ETI in different age groups, such as pediatric population, different genotypes and ETI’s impact on gastrointestinal symptoms.

## Electronic supplementary material

Below is the link to the electronic supplementary material.

Supplementary Material 1

## Data Availability

The datasets used and/or analyzed during the current study are available from the corresponding author on reasonable request.

## Abbreviations

CF: Cystic Fibrosis
CFQ-R: Cystic Fibrosis Questionnaire-Revised
QoL: Quality of Life
ETI: Elexacaftor-Tezacaftor-Ivacaftor
FEV1: Forced Expiratory Volume in 1 s
BMI: Body Mass Index
CFTR: Cystic Fibrosis Transmembrane Conductance Regulator
AIFA: Italian Medicines Agency
