# Correction to: Improved quality of life in cystic fibrosis patients observed up to 36 months after starting Elexacaftor/Tezacaftor/Ivacaftor treatment

**DOI:** 10.1186/s41687-025-00976-0

**Published:** 2026-02-13

**Authors:** Francesca Buniotto, Gloria Tridello, Antonella De Scolari, Ilaria Meneghelli, Emily Pintani, Sandra Perobelli, Marco Cipolli

**Affiliations:** Centro Fibrosi Cistica, AOUI Verona, Piazzale Aristide Stefani, 1, Verona, 37126 Italy

**Correction to: Journal of Patient-Reported Outcomes (2025) 9:48**

10.1186/s41687-025-00879-0

In the original version of this article, the given and family names of authors were incorrectly structured. The name was displayed correctly as below.

Francesca Buniotto, Gloria Tridello, Antonella De Scolari, Ilaria Meneghelli, Emily Pintani, Sandra Perobelli, and Marco Cipolli.

The original version of the article is also updated

