# Supplementary material for: Improved quality of life in cystic fibrosis patients observed up to 36 months after starting Elexacaftor/Tezacaftor/Ivacaftor treatment
Source: J Patient Rep Outcomes. 2025 May 6;9:48. doi: 10.1186/s41687-025-00879-0 (PMC12055669; doi:10.1186/s41687-025-00879-0)
Supplement: Supplementary file 1 — Supplementary Material 1 [file 41687_2025_879_MOESM1_ESM.docx]

**IMPROVED QUALITY OF LIFE IN CYSTIC FIBROSIS PATIENTS OBSERVED UP TO 36 MONTHS AFTER STARTING ELEXACAFTOR/TEZACAFTOR/IVACAFTOR TREATMENT.**

**Part reviewed and added:**

**1. ABSTRACT**

**1.1 (purpose) Page 1, lines 19-20**

This study aims to (fill the data gap in current literature by assessing) evaluate the long-term effects of ETI on QoL.

**1.2 (CFQ-R in methods) and 1.3 (time points) Page2, lines 24-25-26**

Patients were assessed using the CFQ-R (Cystic Fibrosis Questionnaire-Revised). The evaluation time-points were…

**1.3 (type of study design) Page 1, Line 22**

A prospective observational study was conducted…

**(statistical analysis method) Page 2, Line 28**

The Wilcoxon signed-rank test and the Mann-Whitney test were used for the statistical analysis.

**1.4 (Results) Page 2, Lines 30-35**

(We have kept only the most important results but still we have no problem due to the word limit of the abstract)

**1.5 (simplifying language) Pages 1-2, Lines 17-40**

(As suggested we removed redundant words)

**2. BACKGROUND Pages 2-3, Lines 43-71**

We refine the background content, clearly stating the motivation and objectives of the study.

**3. METHODOLOGY**
**3.1** (As suggested, we move that part to the results paragraph) **From lines 90-98 to lines 126-133.**

**3.2** **(inclusion criteria lack of unit of measurement) Page 4, Line 78**

Inclusion criteria were: FEV1 <40% predicted for a minimum of 2 months...

**4. RESULTS
No changes were requested.**

**5. DISCUSSION**
**5.1 (logical order: primary findings, secondary findings and limitations) Lines 249-250,**

The primary aim of our study demonstrated that ETI therapy showed a positive long-term effect on all aspects of QoL covered by the CFQ-R, even in patients with severe disease.

**281-282** The secondary aim of this study was to test influencing factors such as genetics, age and gender in QoL.
**5.2 (more depth exploration of the mechanisms behind certain phenomena, such as the lack of improvement in gastrointestinal symptoms) Lines 269-272**

We did not collect data on this aspect, however it has been already established that ETI is less effective in modifying the pre-existing chronic intestinal inflammation and structural tissue damage in adult patients [13]. This may explain the lack of improvement in GI symptoms and their perception of quality of life.

**Lines 278-279**

Future studies should focus on GI symptoms and QoL also investigating other intestinal aspects such as pancreatic insufficiency.

**(and the influence of genetic status) Lines 283-294**

It is known that PwCF homozygous for F508del generally have a more severe disease and consequently this may influence their QoL.[23, 24]

In our study both groups showed an increase in all domains of CFQ-R over 36 months, independently of their genotype. We only noted a smaller increase in Health Perception and Emotional Functioning in homozygous patients of group B, from baseline to the last timepoint. Similar results were shown in the studies by Di Mango et al.[8] ~~a~~nd Fajac et al. [7].

On the contrary, Carrasco Hernandez et al. [9] identified a difference between heterozygous and homozygous subjects, with the latter showing a greater decrease in CFQ-R scores after 12 months. The authors did not provide explanation on this result. We think that the persistence of a better clinical status may be the principal factor influencing QoL, regardless of the genetic defect. In general, the perception of QoL depends on many factors, including the acceptance and the adaptation to the disease, family environment and best supportive care [1].

**6. CONCLUSION**
**6.1**  **(simplifying language) Lines 350-352**

ETI treatment significantly improved patients’ QoL in both groups. These improvements were maintained over long-term follow-up (36 months). For both groups, the changes identified at the last follow-up showed no major differences by gender, age or genetic status.
**6.2 (directions for future research)** **Lines 353-355**

It will be interesting to explore in future research the long-term effects of ETI in different age groups, such as pediatric population, different genotypes and ETI’s impact on gastrointestinal symptoms.
